# Supplementary material for: What does it take to learn the rules of RNA base pairing? A lot less than you may think
Source: bioRxiv. 2025 Aug 2:2025.07.31.668042. Preprint. [Version 1] doi: 10.1101/2025.07.31.668042 (PMC12324431; doi:10.1101/2025.07.31.668042)
Supplement: Supplement 1 [file media-1.gz › supplemental_material/experiments/g6_optimize_param_TORNADO_7Fam.len0_400.nsq400_uniform/trna1415_annote_2of2.noss.mea/acc_found_i26.pdf]

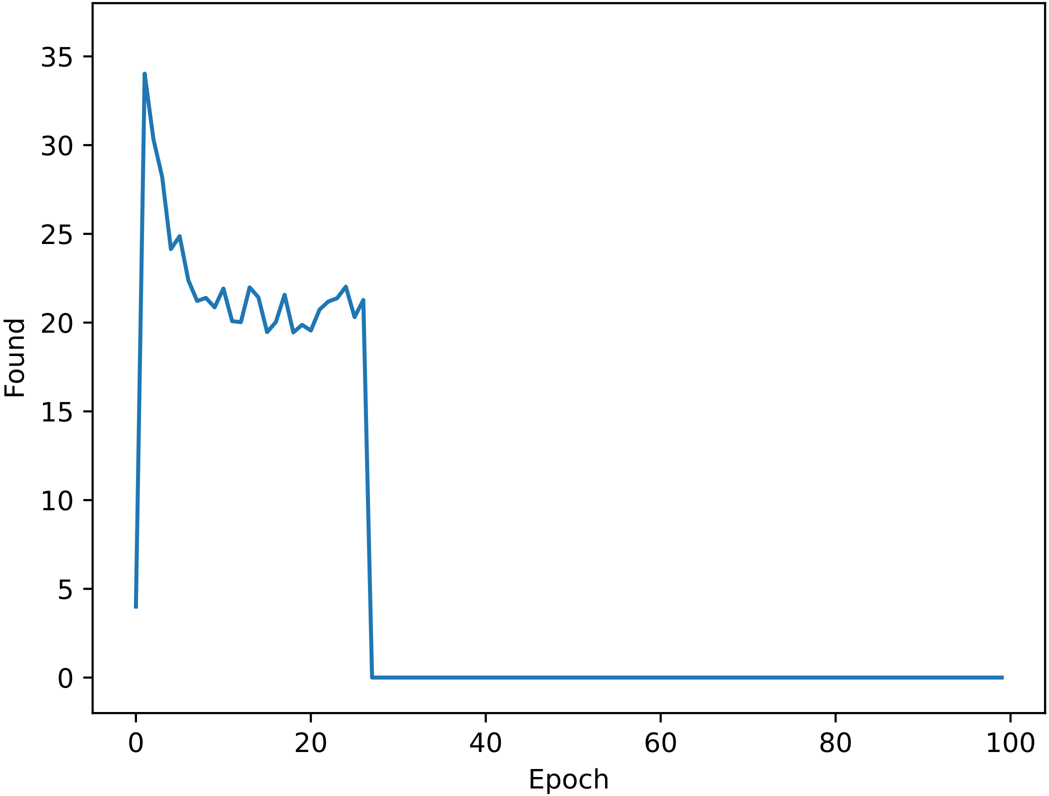

35
30
25
20
15
10
5
0
Found
0
20
40
60
80
100
Epoch
